# Crystal structure, Hirshfeld surface analysis, calculations of crystal voids, inter­action energy and energy frameworks as well as density functional theory (DFT) calculations of 3-[2-(morpholin-4-yl)eth­yl]-5,5-di­phenyl­imidazolidine-2,4-dione

**DOI:** 10.1107/S2056989024002445

**Published:** 2024-03-26

**Authors:** Houda Lamssane, Amal Haoudi, Badr Eddine Kartah, Ahmed Mazzah, Joel T. Mague, Tuncer Hökelek, Youssef Kandri Rodi, Nada Kheira Sebbar

**Affiliations:** a Laboratory of Applied Organic Chemistry, Sidi Mohamed Ben Abdellah University, Faculty Of Science And Technology, Road Immouzer, BP 2202 Fez, Morocco; b Laboratory of Plant Chemistry, Organic and Bioorganic Synthesis, Faculty of Sciences, Mohammed V University in Rabat, 4 Avenue Ibn Battouta BP 1014 RP, Morocco; c Science and Technology of Lille USR 3290, Villeneuve d’ascq cedex, France; d Department of Chemistry, Tulane University, New Orleans, LA 70118, USA; e Department of Physics, Hacettepe University, 06800 Beytepe, Ankara, Türkiye; f Laboratory of Organic and Physical Chemistry, Applied Bioorganic Chemistry Team, Faculty of Sciences, Ibnou Zohr University, Agadir, Morocco; Vienna University of Technology, Austria

**Keywords:** crystal structure, imidazolidinedione, hydrogen bond, C—H⋯π(ring) inter­action

## Abstract

In the title mol­ecule, C_21_H_23_N_3_O_3_, the imidazolidine ring slightly deviates from planarity and the morpholine ring exhibits the chair conformation. In the crystal, N—H⋯O and C—H⋯O hydrogen bonds form helical chains of mol­ecules extending parallel to the *c* axis that are connected by C—H⋯π(ring) inter­actions. A Hirshfeld surface analysis reveals that the most important contributions for the crystal packing are from H⋯H (55.2%), H⋯C/C⋯H (22.6%) and H⋯O/O⋯H (20.5%) inter­actions. The volume of the crystal voids and the percentage of free space were calculated to be 236.78 Å^3^ and 12.71%, respectively. Evaluation of the electrostatic, dispersion and total energy frameworks indicates that the stabilization is dominated by the nearly equal electrostatic and dispersion energy contributions. The DFT-optimized mol­ecular structure at the B3LYP/6-311 G(d,p) level is compared with the experimentally determined mol­ecular structure in the solid state. Moreover, the HOMO–LUMO behaviour was elucidated to determine the energy gap.

## Abstract 2

In the title mol­ecule, the imidazolidine ring slightly deviates from planarity and the morpholine ring exhibits the usual chair conformation. In the crystal, N—H⋯O and C—H⋯O hydrogen bonds form helical chains of mol­ecules extending parallel to the *c* axis that are connected by C—H⋯π(ring) inter­actions into a tri-periodic network.

## 1. Chemical context

The investigation of compounds comprising the hydantoin moiety has gained inter­est in the quest for the development of new drugs because of their similarity to natural amino acids (Śladowska *et al.*, 2016 ▸). Such compounds show various pharmacological properties, including anti­bacterial (Pandeya *et al.*, 2000 ▸; Sangeetha *et al.*, 2016 ▸), anti­convulsant (Emami *et al.*, 2021 ▸), anti­diabetic (Salem *et al.*, 2018 ▸), anti­tumor (Żesławska *et al.*, 2021 ▸), anti­nociceptive and anti-inflammatory (Abdel-Aziz *et al.*, 2016 ▸; da Silva Guerra *et al.*, 2011 ▸) activities. One of the foremost pharmaceutical drugs in the hydantoin class is phenytoin, also known as 5,5-di­phenyl­hydantoin (systematic name: 5,5-di­phenyl­imidazolidine-2,4-dione). This compound and its derivatives are considered to be potential pharmaceutical agents due to their extended shelf life. However, their optimum efficacy depends on how easily they break down in the body (Al-Nuzal *et al.*, 2018 ▸). With respect to the biological importance of phenytoin, we were inter­ested in the synthesis of a new derivative thereof, *viz*. 3-[2-(morpholin-4-yl)eth­yl]-5,5-di­phenyl­imidazolidine-2,4-di­one, (**I**), C_21_H_23_N_3_O_3_, through an alkyl­ation reaction under the conditions of phase transfer catalysis. We report here the mol­ecular and crystal structures as well as the Hirshfeld surface analysis of this compound, as well as inter­molecular inter­action energies, energy frameworks, and a comparison of the experimentally determined mol­ecular structure in the solid state with that of an optimized structure obtained by density functional theory (DFT). 
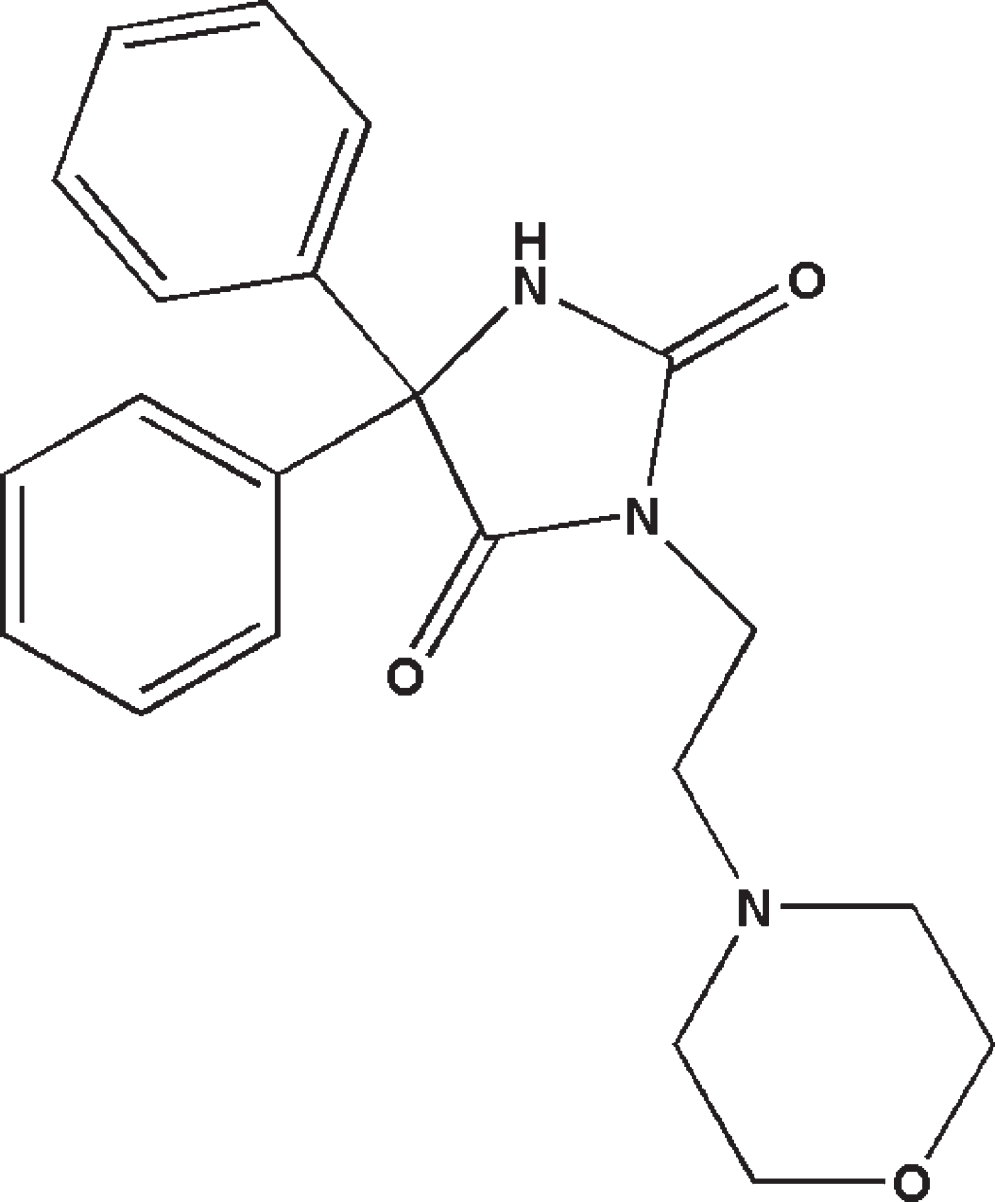


## 2. Structural commentary

**Figure 1:**
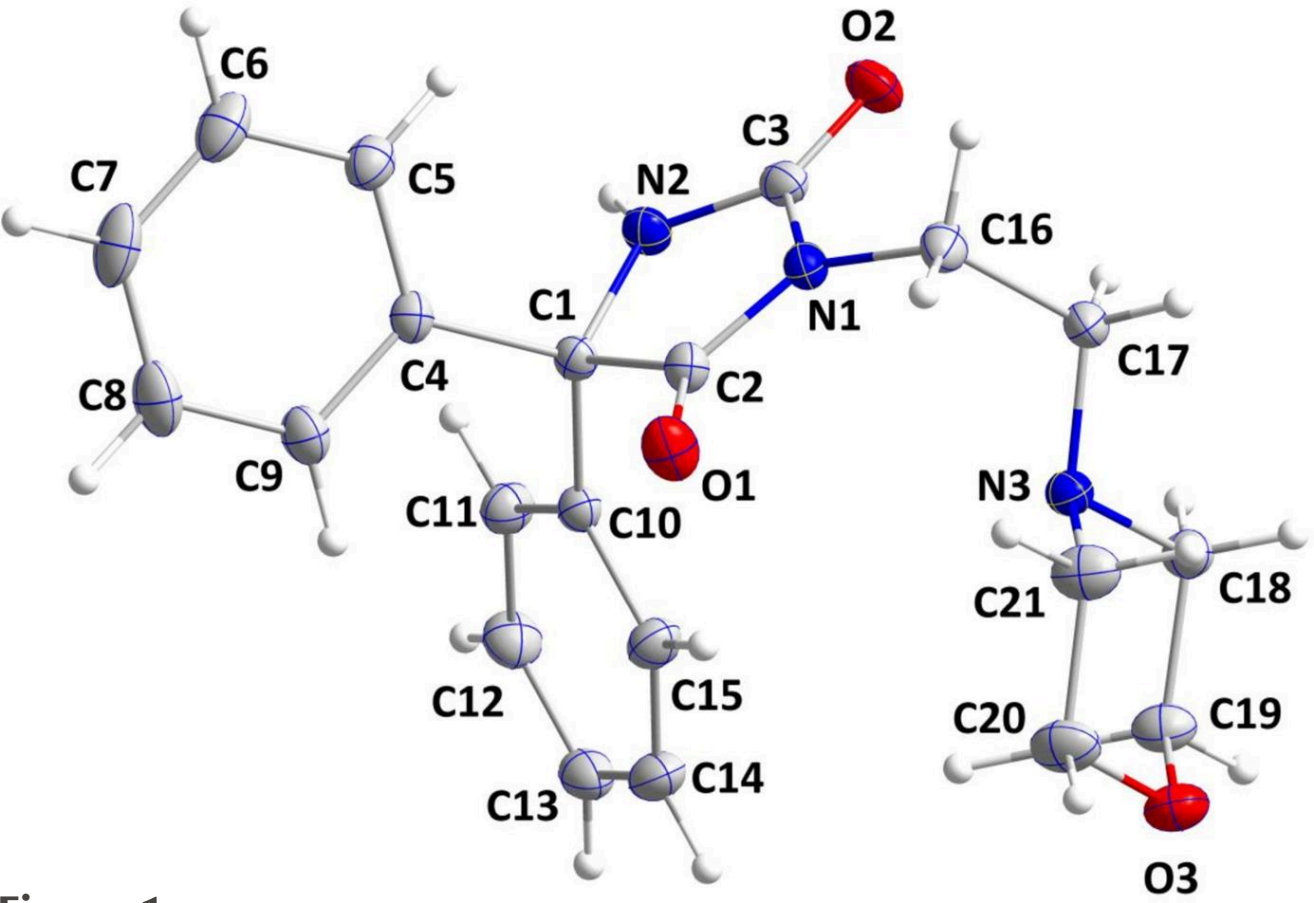
The title mol­ecule with the labelling scheme and displacement ellipsoids drawn at the 50% probability level.

The mol­ecular structure of (**I**) is displayed in Fig. 1 ▸. The imidazolidine ring deviates from planarity (root-mean-square deviation of the fitted atoms = 0.0273 Å) with atoms C1 and N2 being displaced by 0.0359 (7) and −0.0359 (7) Å, respectively, from the mean plane. The C4–C9 and C10–C15 benzene rings are inclined to the above plane by 76.55 (4) and 65.07 (4)°, respectively. The N1—C2, N1—C3 and N2—C3 distances are 1.367 (2), 1.406 (2) and 1.348 (2) Å. The sums of the bond angles about N1 and N2 of 360.0 and 359° indicate that the lone electron pairs at the nitro­gen atoms are involved in N→C π bonding. The morpholine ring adopts a chair conformation [puckering parameters (Cremer & Pople, 1975 ▸): *Q* = 0.5831 (14) Å, θ = 176.90 (14)°, φ = 339 (3)°].

## 3. Supra­molecular features

**Figure 2:**
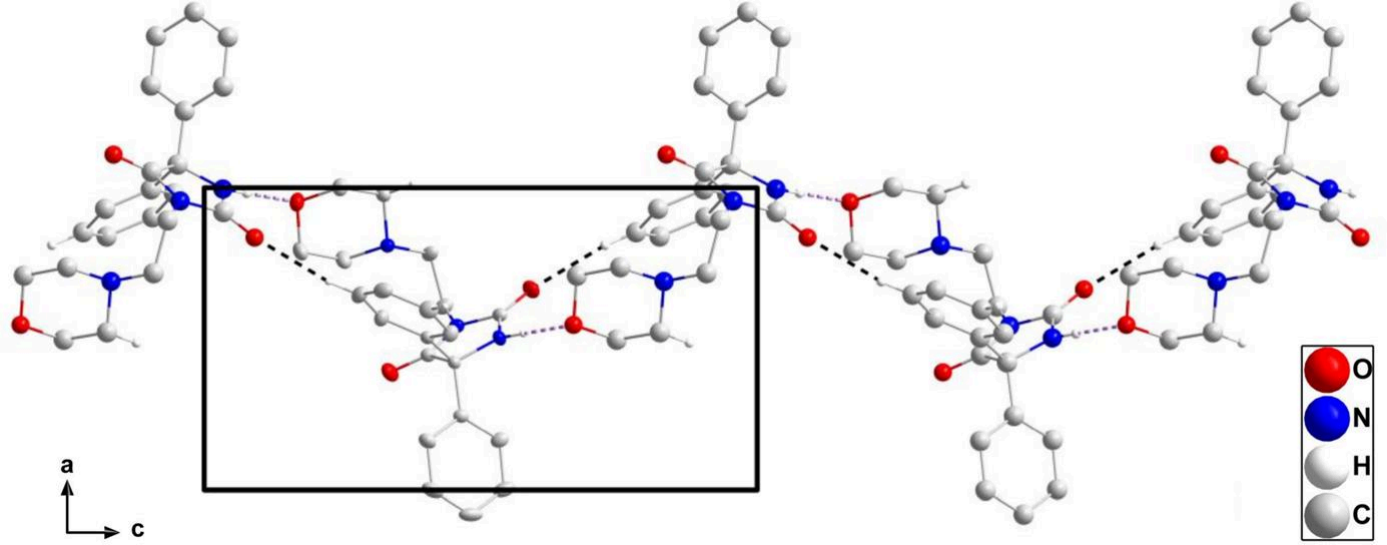
A portion of one helical chain in the crystal structure viewed along the *b* axis. N—H⋯O and C—H⋯O hydrogen bonds are depicted by violet and black dashed lines, respectively. Non-inter­acting hydrogen atoms are omitted for clarity.

**Figure 3:**
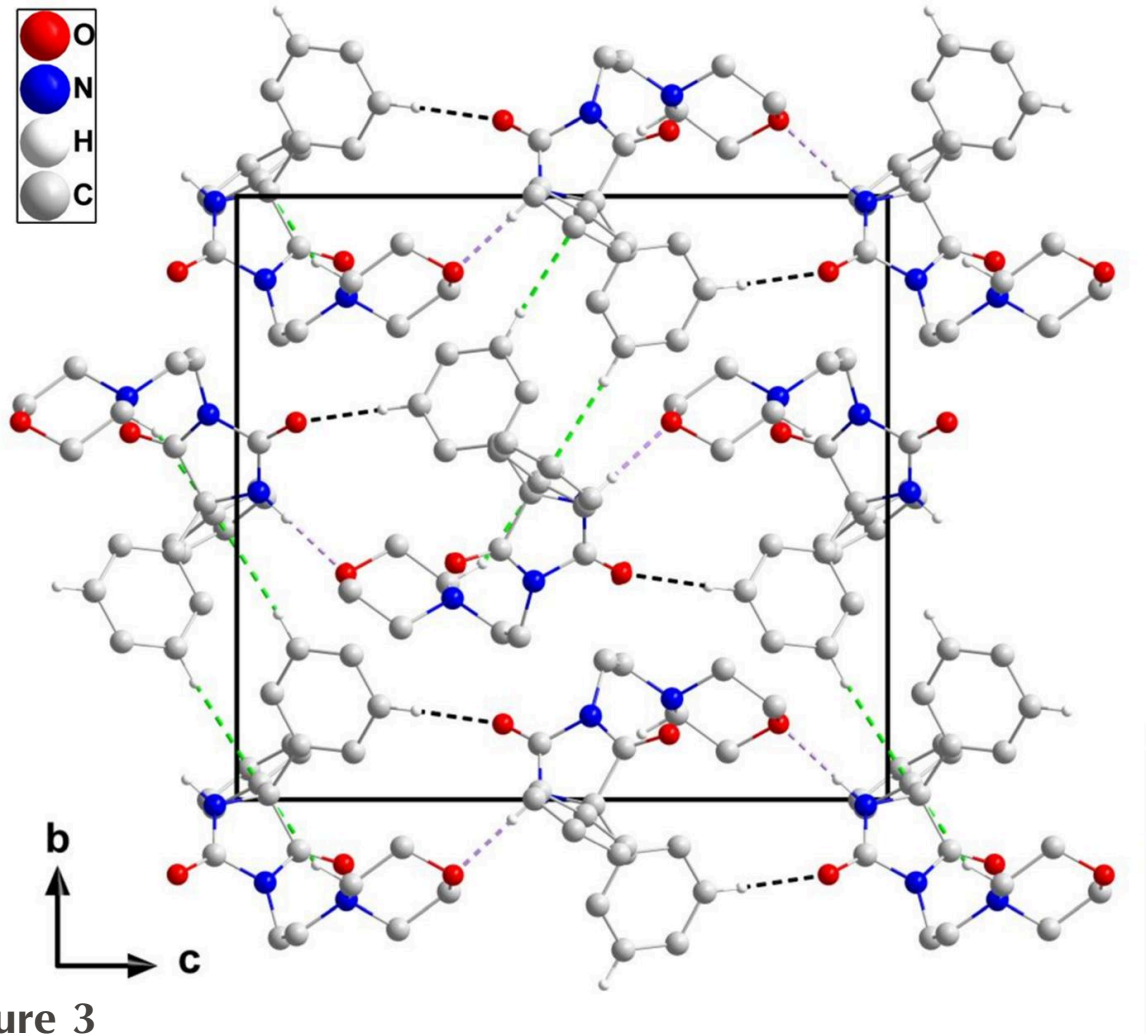
The crystal packing viewed along the *a* axis with N—H⋯O and C—H⋯O hydrogen bonds depicted by violet and black dashed lines, respectively, and with C—H⋯π(ring) inter­actions depicted by green dashed lines. Non-inter­acting hydrogen atoms are omitted for clarity.

In the crystal of (**I**), N2—H2⋯O1 and C14—H14⋯O2 hydrogen bonds (Table 1 ▸) form helical chains of mol­ecules extending parallel to the *c* axis (Fig. 2 ▸). Individual mol­ecules are connected by weak C12—H12⋯*Cg*3 and C18—H18*B*⋯*Cg*3 inter­actions (Table 1 ▸) into a tri-periodic network (Fig. 3 ▸), which appears to have small pores in agreement with the calculation of a relatively small void space (*vide infra*).

## 4. Hirshfeld surface analysis

**Figure 4:**
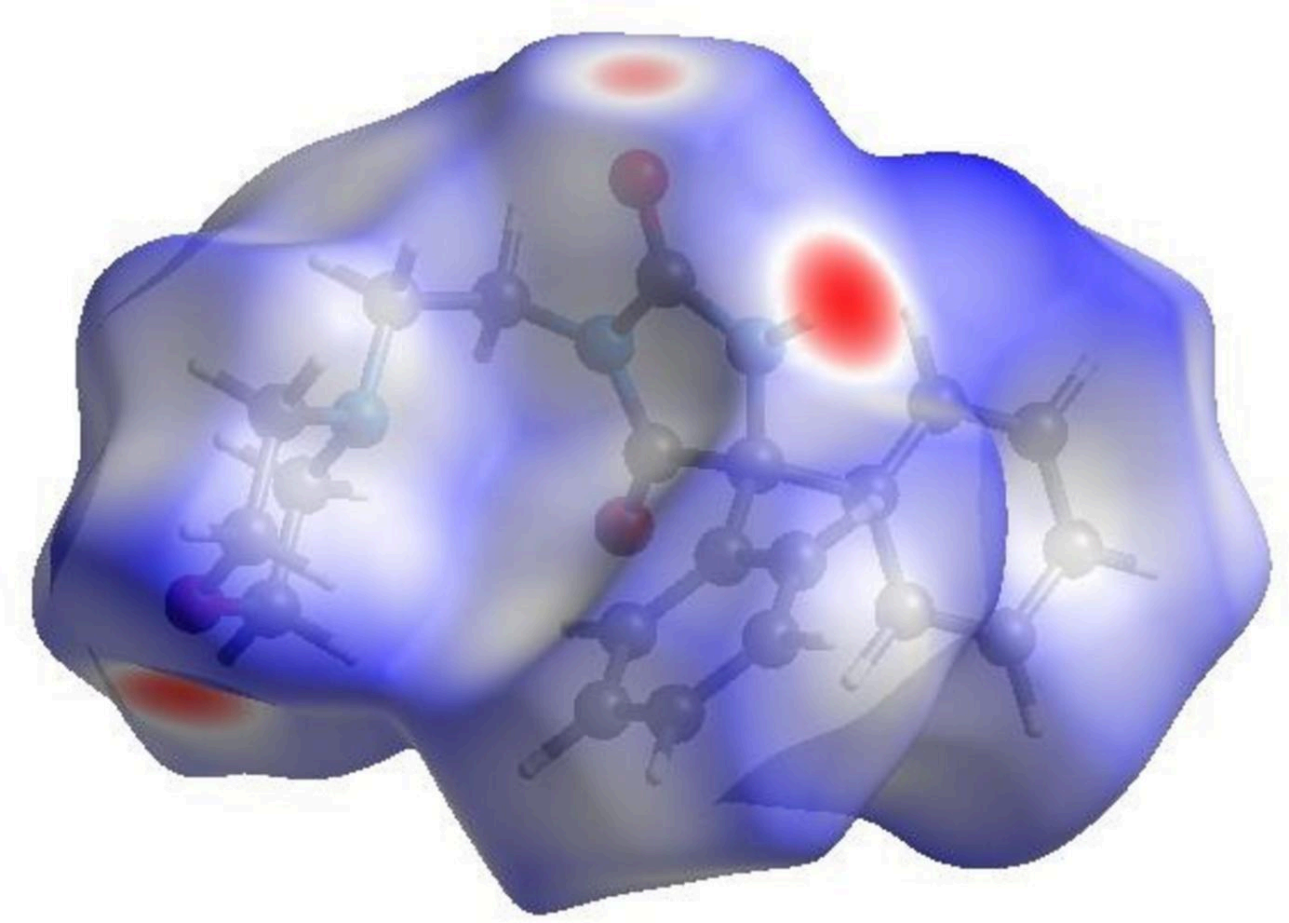
View of the three-dimensional Hirshfeld surface of (**I**) plotted over *d*_norm_.

**Figure 5:**
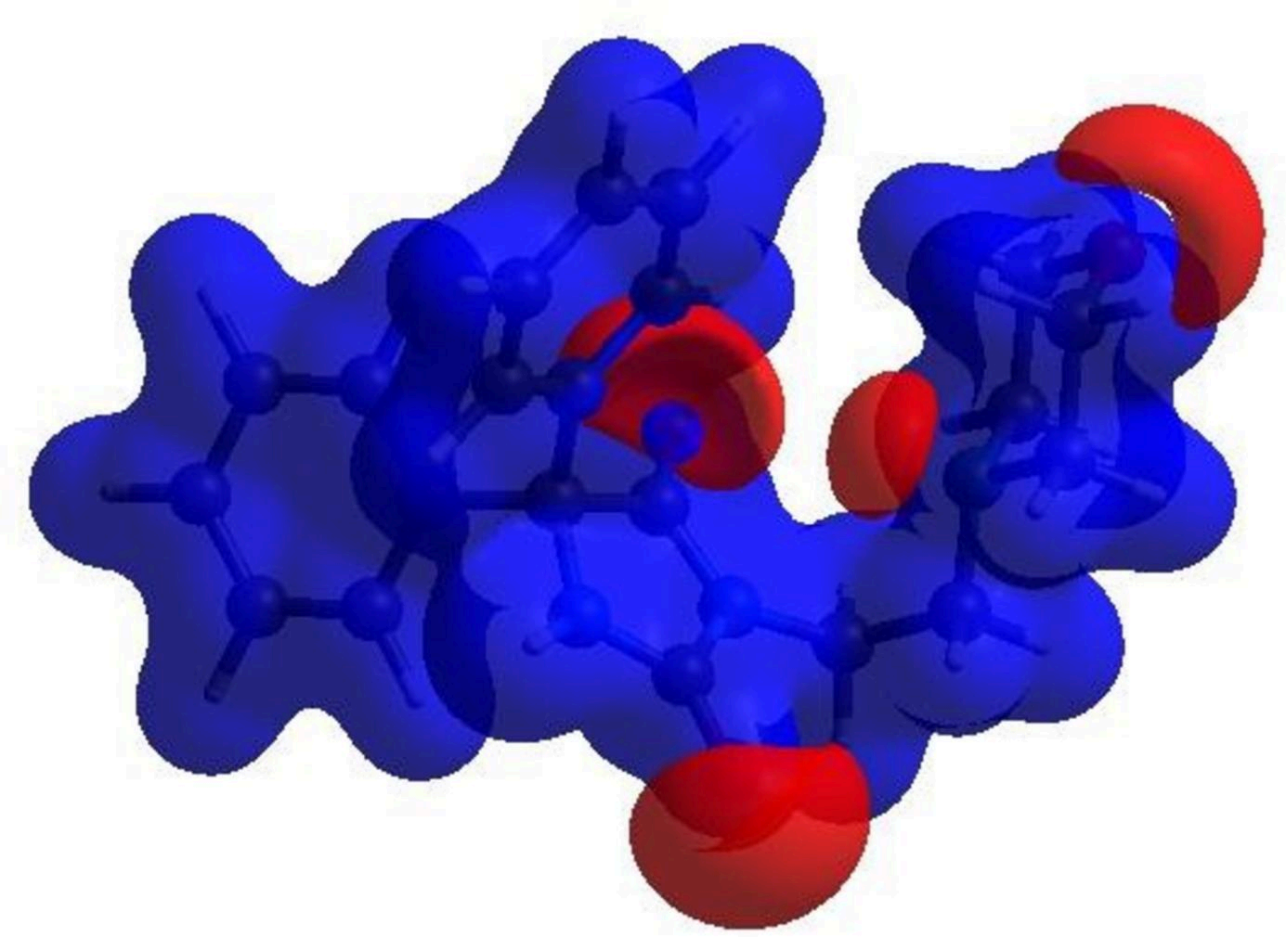
View of the three-dimensional Hirshfeld surface of (**I**) plotted over electrostatic potential energy using the STO-3 G basis set at the Hartree–Fock level of theory. Hydrogen-bond donors and acceptors are shown as blue and red regions around the atoms corresponding to positive and negative potentials, respectively.

**Figure 6:**
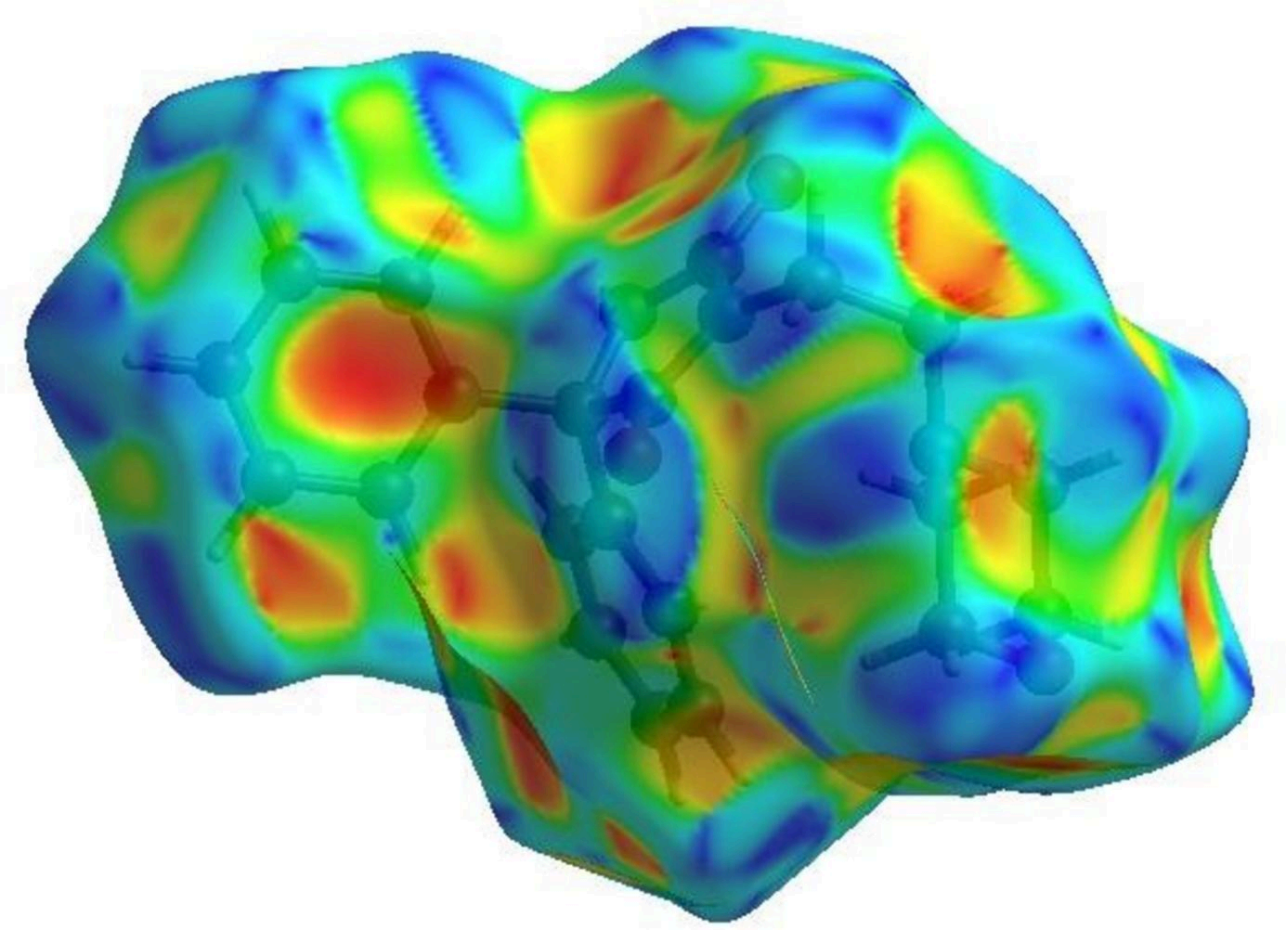
Hirshfeld surface of the title compound plotted over shape-index.

**Figure 7:**
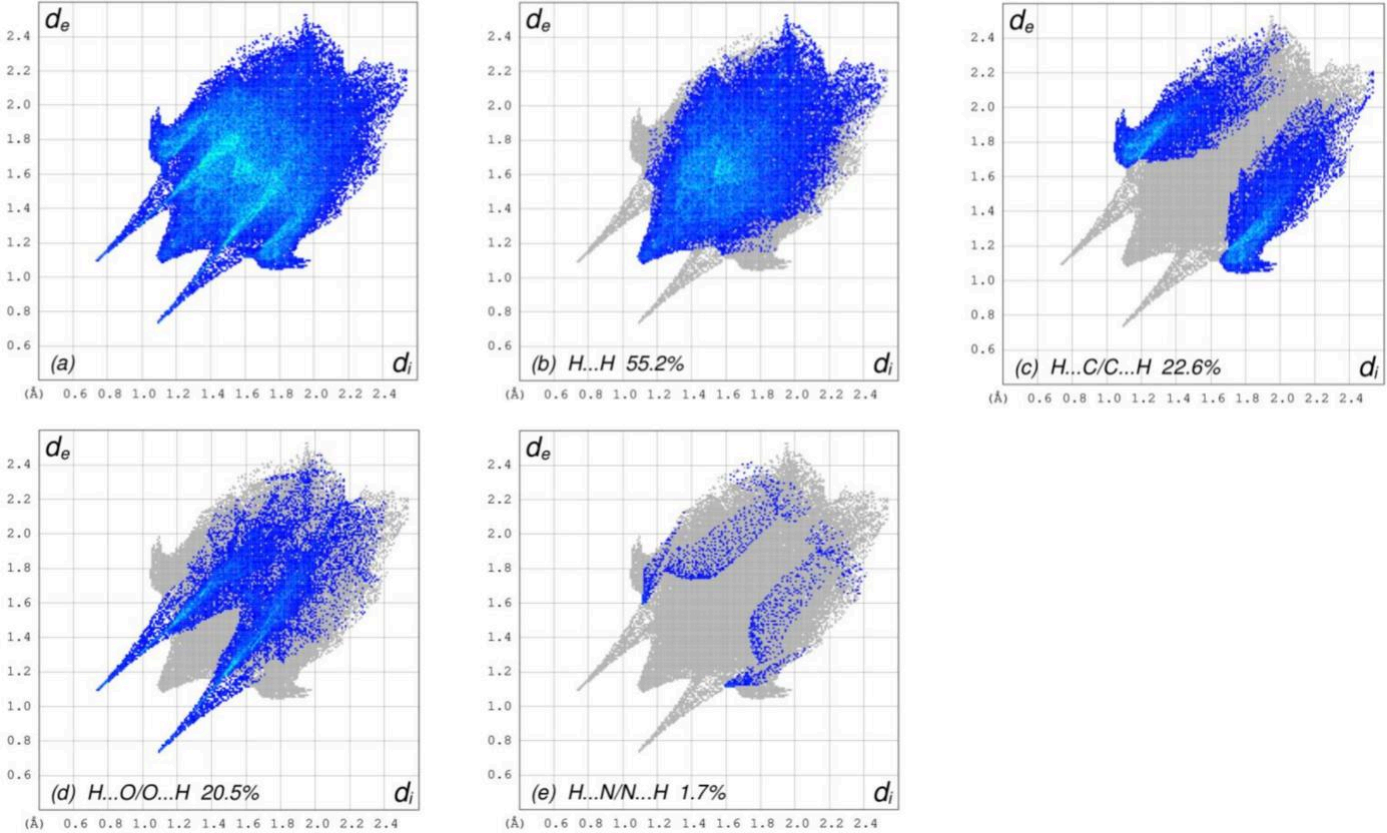
The full two-dimensional fingerprint plots for (**I**), showing (*a*) all inter­actions, and delineated into (*b*) H⋯H, (*c*) H⋯C/C⋯H, (*d*) H⋯O/O⋯H and (*e*) H⋯N/N⋯H inter­actions. The *d*_i_ and *d*_e_ values are the closest inter­nal and external distances (in Å) from given points on the Hirshfeld surface.

In order to visualize the inter­molecular inter­actions in the crystal of (**I)**, a Hirshfeld surface (HS) analysis (Hirshfeld, 1977 ▸; Spackman & Jayatilaka, 2009 ▸) was carried out using *CrystalExplorer* (Spackman *et al.*, 2021 ▸). In the HS plotted over *d*_norm_ (Fig. 4 ▸), the white surface indicates contacts with distances equal to the sum of van der Waals radii and the red and blue colours indicate distances shorter (in close contact) or longer (distant contact) than the sum of the van der Waals radii, respectively (Venkatesan *et al.*, 2016 ▸). The bright-red spots on the surface indicate the roles of adjacent atoms as the respective donors and/or acceptors and they also appear as blue and red regions corresponding to positive (hydrogen-bond donors) and negative (hydrogen-bond acceptors) potentials on the HS mapped over electrostatic potential (Spackman *et al.*, 2008 ▸; Jayatilaka *et al.*, 2005 ▸), as shown in Fig. 5 ▸. The shape-index of the HS is a tool to visualize *π*–*π* stacking by the presence of adjacent red and blue triangles. However, Fig. 6 ▸ clearly suggests that there are no *π*–*π* inter­actions in the crystal structure of (**I**). The overall two-dimensional fingerprint plot, Fig. 7 ▸ *a*, and those delineated into H⋯H, H⋯C/C⋯H, H⋯O/O⋯H and H⋯N/N⋯H (McKinnon *et al.*, 2007 ▸) are illustrated in Fig. 7 ▸ *b*–*e*, respectively, together with their relative contributions to the Hirshfeld surface. The most abundant inter­action is H⋯H, contributing with 55.2% to the overall crystal packing, which is reflected in Fig. 7 ▸ *b* as widely scattered points of high density due to the large hydrogen content with the tip at *d*_e_ = *d*_i_ = 1.10 Å. The H⋯C/C⋯H contacts, contributing with 22.6% to the overall crystal packing, are shown in Fig. 7 ▸ *c* with the tips at *d*_e_ + *d*_i_ = 2.74 Å and are attributed to C—H⋯π inter­actions. The symmetrical pair of spikes in the fingerprint plot delineated into H⋯O/O⋯H contacts with the tips at *d*_e_ + *d*_i_ = 1.84 Å (Fig. 7 ▸ *d*) make a 20.5% contribution to the HS. Finally, the tiny pair of spikes with the tips at *d*_e_ + *d*_i_ = 2.70 Å in the fingerprint plot delineated into H⋯N/N⋯H contacts (Fig. 7 ▸ *e*) contributes only 1.7% to the HS.

**Figure 8:**
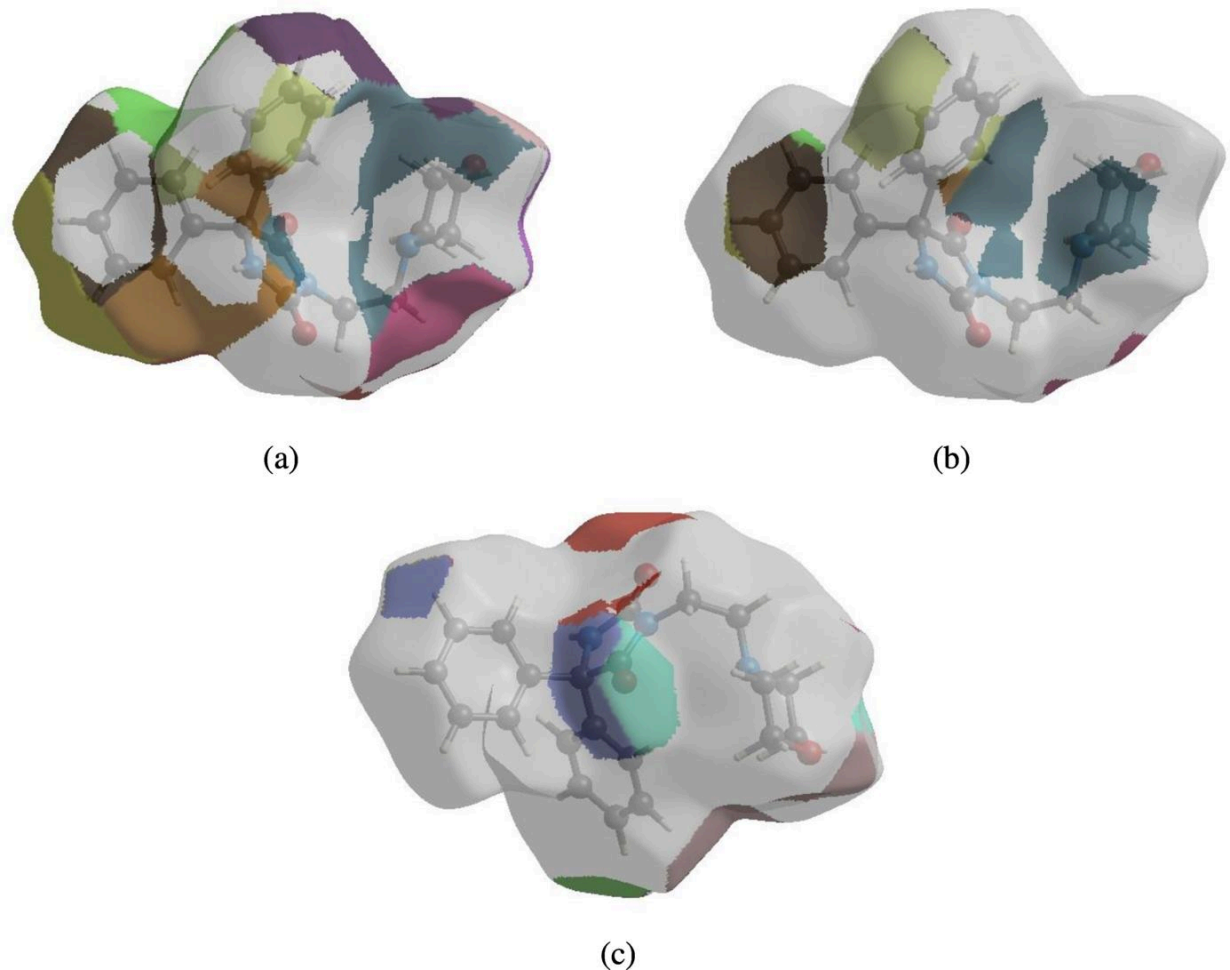
Hirshfeld surface patches plotted onto the surface for (*a*) H⋯H, (*b*) H⋯C/C⋯H and (*c*) H⋯O/O⋯H inter­actions.

The nearest neighbour coordination environment of a mol­ecule can be determined from the colour patches on the HS based on how close to other mol­ecules they are. The Hirshfeld surface representations with the function *d*_norm_ plotted onto the surface are shown for the H⋯H, H⋯C/C⋯H and H⋯O/O⋯H inter­actions in Fig. 8 ▸ *a*–*c*. The Hirshfeld surface analysis confirms the importance of H-atom contacts in establishing the packing. The large number of H⋯H, H⋯C/C⋯H and H⋯O/O⋯H inter­actions suggest that van der Waals inter­actions and hydrogen-bonding inter­actions play the major roles in the crystal packing (Hathwar *et al.*, 2015 ▸).

## 5. Crystal voids

**Figure 9:**
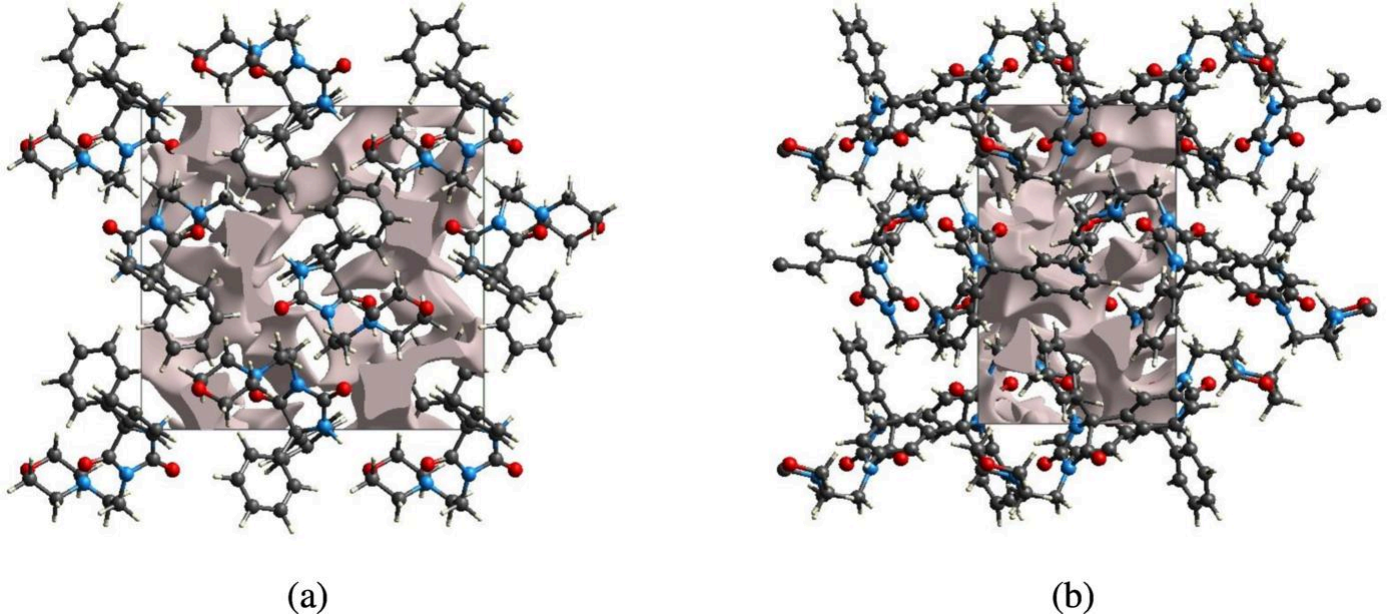
Graphical views of voids in the crystal packing of (**I**), (*a*) along the *a* axis and (*b*) along the *c* axis.

The strength of the crystal packing is important for determining the response to an applied mechanical force. If significant voids are present in the crystal, the mol­ecules are not tightly packed and a small amount of applied external mechanical force may easily break the crystal. To check the mechanical stability of the crystal, a void analysis was performed by adding up the electron densities of the spherically symmetric atoms contained in the asymmetric unit (Turner *et al.*, 2011 ▸). The void surface is defined as an isosurface of the procrystal electron density and is calculated for the whole unit cell where the void surface meets the boundary of the unit cell, and capping faces are generated to create an enclosed volume. The volume of the crystal voids (Fig. 9 ▸ *a*,*b*) and the percentage of free space in the unit cell were calculated as 236.78 Å^3^ and 12.71%.

## 6. Inter­action energy calculations and energy frameworks

**Figure 10:**
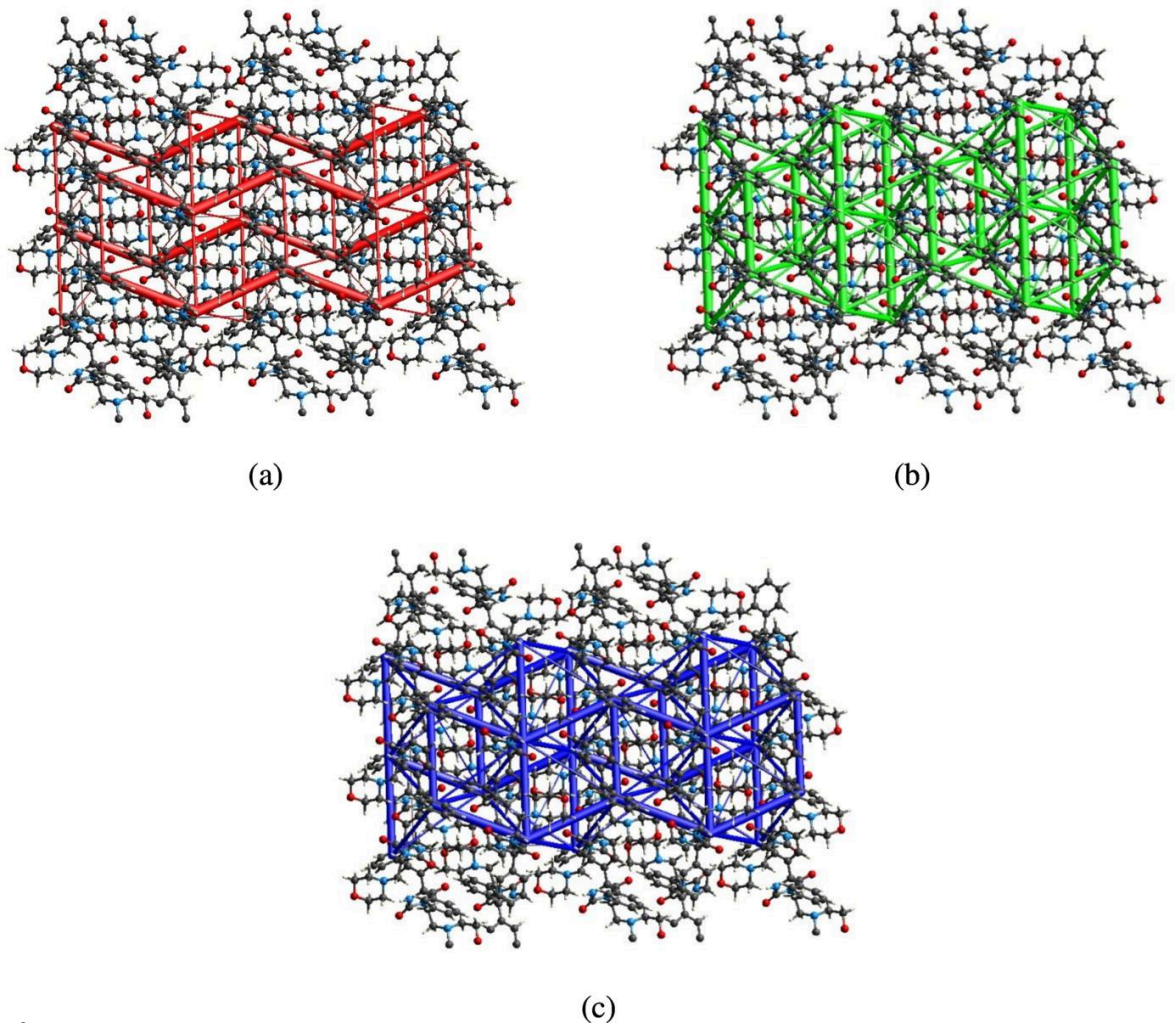
The energy frameworks for a cluster of mol­ecules of (**I**) viewed down the *b* axis, showing (*a*) electrostatic energy, (*b*) dispersion energy and (*c*) total energy diagrams. The cylindrical radius is proportional to the relative strength of the corresponding energies and they were adjusted to the same scale factor of 80 with cut-off value of 5 kJ mol-1 within 2×2×2 unit cells.

The inter­molecular inter­action energies were calculated using the CE–B3LYP/6–31G(d,p) energy model available in *CrystalExplorer* (Spackman *et al.*, 2021 ▸) where a cluster of mol­ecules is generated by applying crystallographic symmetry operations with respect to a selected central mol­ecule within a radius of 3.8 Å (Turner *et al.*, 2014 ▸). The total inter­molecular energy (*E*_tot_) is the sum of electrostatic (*E*_ele_), polarization (*E*_pol_), dispersion (*E*_dis_) and exchange-repulsion (*E*_rep_) energies (Turner *et al.*, 2015 ▸) with scale factors of 1.057, 0.740, 0.871 and 0.618, respectively (Mackenzie *et al.*, 2017 ▸). Hydrogen-bonding inter­action energies (in kJ mol^−1^) were calculated to be −50.2(*E*_ele_), −12.7 (*E*_pol_), −27.6 (*E*_dis_), 58.2 (*E*_rep_) and −50.5 (*E*_tot_) for N2—H2⋯O3 and −17.4 (*E*_ele_), −3.7 (*E*_pol_), −56.2 (*E*_dis_), 43.2 (*E*_rep_) and −43.4 (*E*_tot_)] for C14—H14⋯O2. Energy frameworks combine the calculation of inter­molecular inter­action energies with a graphical representation of their magnitude (Turner *et al.*, 2015 ▸). Energies between mol­ecular pairs are represented as cylinders joining the centroids of pairs of mol­ecules with the cylinder radius proportional to the relative strength of the corresponding inter­action energy. Energy frameworks were constructed for *E*_ele_ (red cylinders), *E*_dis_ (green cylinders) and *E*_tot_ (blue cylinders) and are displayed in Fig. 10 ▸ *a*–*c*. The evaluation of the electrostatic, dispersion and total energy frameworks indicate that the stabilization is dominated by nearly equal electrostatic and dispersion energy contributions in the crystal structure of (**I**).

## 7. DFT calculations

**Figure 11:**
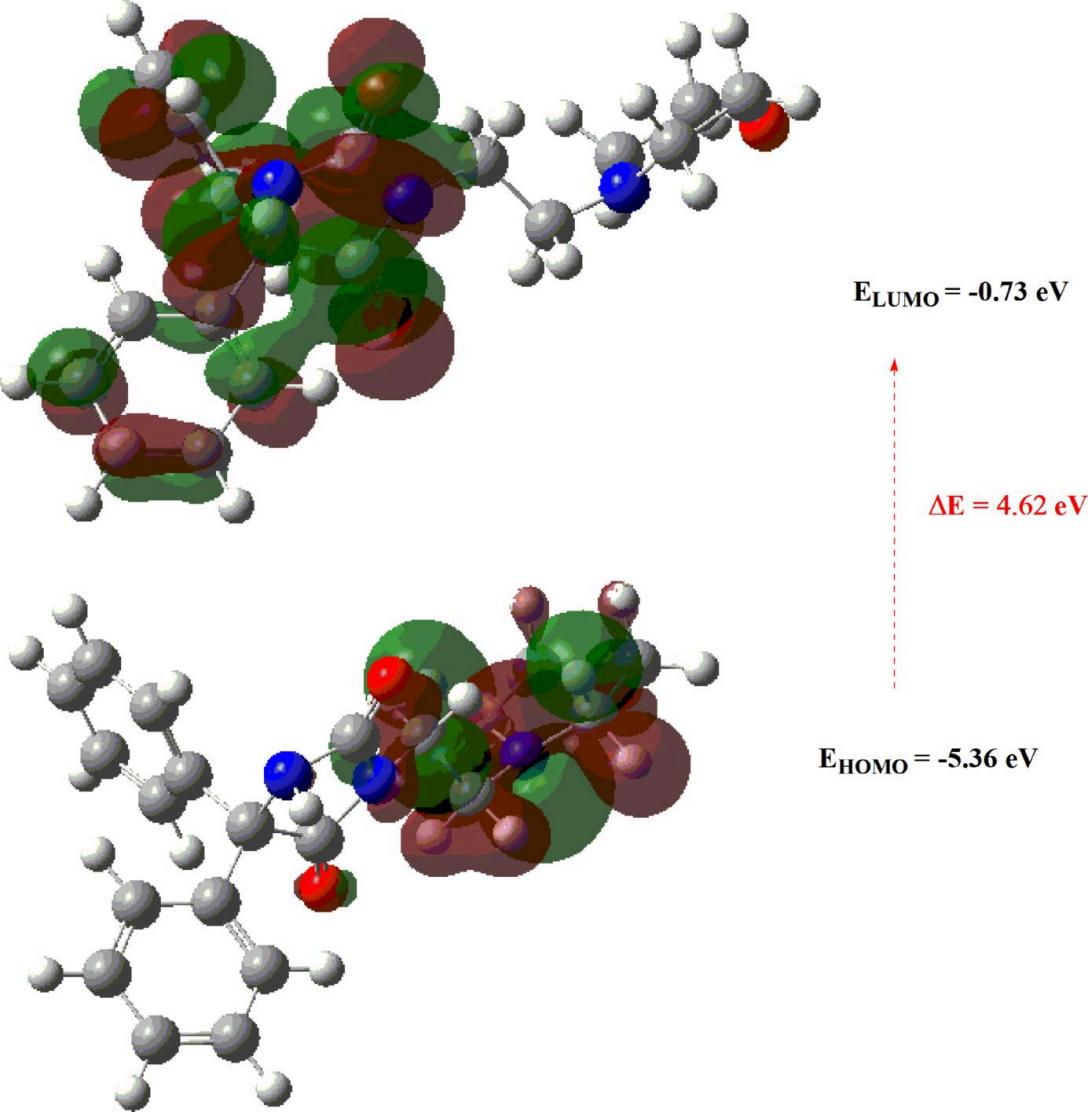
The energy band gap of the title compound.

The optimization of the mol­ecular gas-phase structure of (**I**) was conducted using density functional theory (DFT) with the B3LYP functional and 6-311G(d,p) basis-set calculations, as implemented in *GAUSSIAN09* (Becke, 1993 ▸; Frisch *et al.*, 2009 ▸). The comparison between theoretical and experimental results revealed good agreement (Table 2 ▸). Essential parameters, such as the highest-occupied mol­ecular orbital (HOMO) serving as an electron donor and the lowest-unoccupied mol­ecular orbital (LUMO) acting as an electron acceptor, were examined, whereby a small energy gap indicates high mol­ecular polarizability and reactivity. Numerical parameters of *E*_HOMO_, *E*_LUMO_, electronegativity (χ), hardness (η), potential (μ), electrophilicity (ω), and softness (σ) are detailed in Table 3 ▸. The values of χ and η are particularly significant for assessing both reactivity and stability. The electron transition from the HOMO to the LUMO energy level is shown in Fig. 11 ▸. Both the HOMO and LUMO are localized in the mol­ecular plane. The energy band gap (*E* = *E*_LUMO_ − *E*_HOMO_) is approximately 4.62 eV, with frontier mol­ecular orbital (FMO) energies, *E*_HOMO_ and *E*_LUMO_, determined as −5.36 and −0.73 eV, respectively.

## 8. Database survey

**Figure 12:**
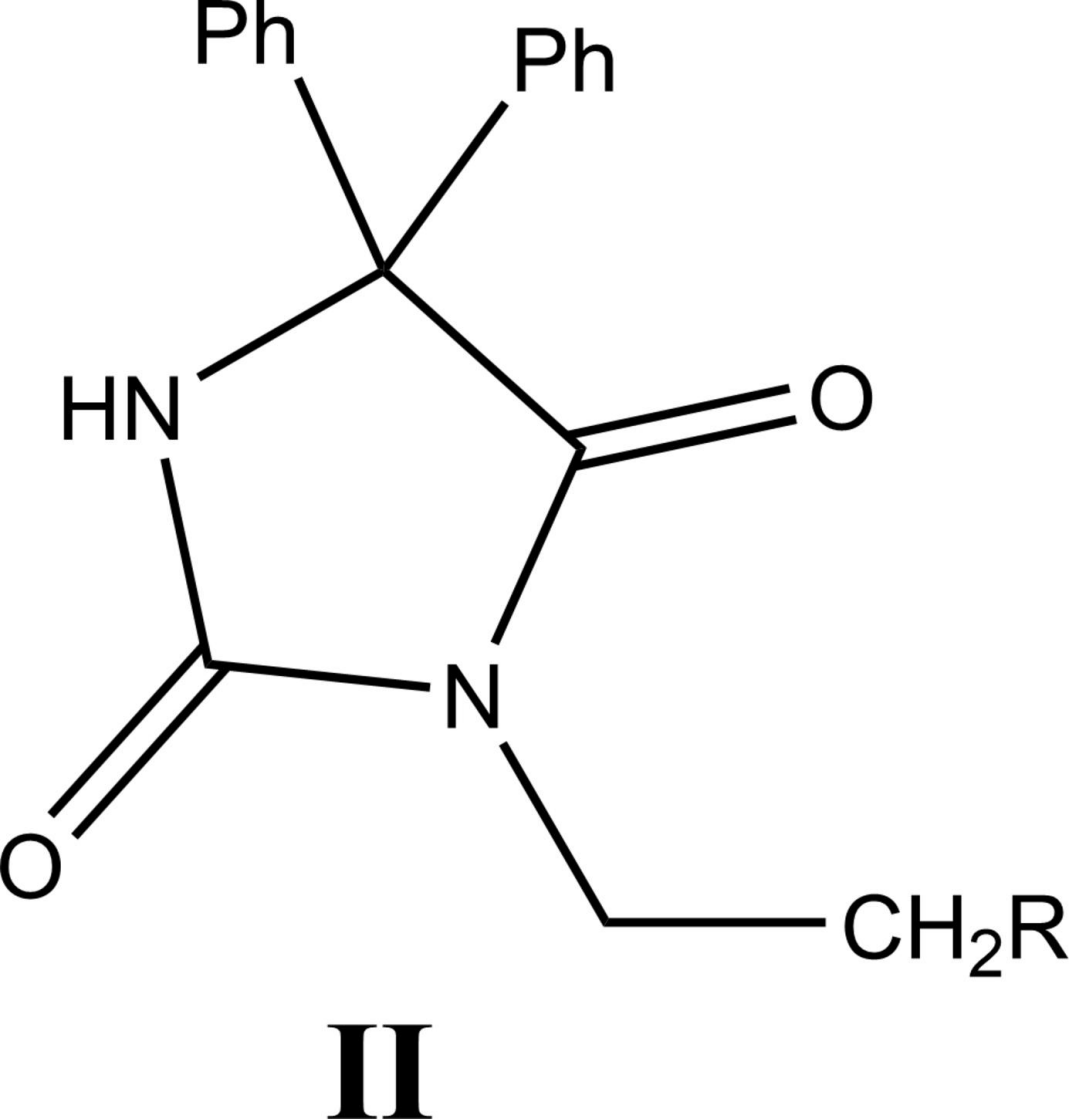
The mol­ecular moiety used for the CSD search procedure.

A search of the Cambridge Structural Database (CSD; Groom *et al.*, 2016 ▸; updated January 2024) with the fragment (**II**) (Fig. 12 ▸) gave ten hits, one of which (LUHFID; Ooms *et al.*, 2002 ▸) is the same as (**I**) except for having a bromine atom at the 4-position of each phenyl ring. Here, the C—N distances in the ring are similar, indicating delocalization of the π electrons. Although the morpholine ring also adopts a chair conformation, the large displacement ellipsoids for the constituent atoms indicate at least considerable librational motion and likely some degree of positional disorder. The remaining matches have *R* = H (FEHPUG; Guerrab *et al.*, 2017*a* ▸), Me (WEMQUD; Guerrab *et al.*, 2017*c* ▸ and WEMQUD01; Trišovic *et al.*, 2019 ▸), Et (QENBET; Guerrab *et al.*, 2018*a* ▸), –(CH_2_)_2_Me (GEMSOG; Guerrab *et al.*, 2017*b* ▸), –(CH_2_)_3_Me (QENBOD; Guerrab *et al.*, 2018*b* ▸), –(CH_2_)_5_Me (QAGPAT; Guerrab *et al.*, 2020 ▸), –(CH_2_)_7_Me (PAJMAS; Guerrab *et al.*, 2022 ▸) and Br (NIBMOE; Guerrab *et al.*, 2023 ▸). In all cases, the five-membered ring is close to planarity and the nitro­gen lone pairs are involved in N→C π bonding.

## 9. Synthesis and crystallization

In a flask, phenytoin (0.5 g, 1.98 mmol) was mixed with 4-(2-chloro­eth­yl)morpholine hydro­chloride (0.37 g, 1.01 mmol) in DMF (20 ml) in the presence of potassium carbonate (0.41 g, 2.96 mmol) and tetra-*n*-butyl­ammonium bromide (BTBA, 0.07 g, 0.22 mmol). The reaction mixture was agitated for 48 h at room temperature, followed by removal of the solvent under reduced pressure. The residue was purified by recrystallization from methanol. Yield: 85%, *R*_f_: 0.17 (ethyl acetate/hexa­ne: 1/2), m.p. 363 K, LCMS (ESI): 366.1812 [*M*+H^+^], ^1^H NMR (CDCl_3_-300 MHz): δ (ppm) 7.36–7.47 (*m*, 10H, H_Ar_); 7.08 (*s*, 1H, NH), 3.72 (*t*, 2H, CH_2_, ^3^ *J*_H–H_ = 6 MHz), 3.60 (*t*, 4H, CH_2_, ^3^ *J*_H–H_ = 6 MHz), 2.65 (*t*, 2H, CH_2_, ^3^ *J*_H–H_ = 6 MHz), 2.50 (*t*, 4H, CH_2_, ^3^ *J*_H–H_ = 6 MHz), ^13^C NMR (CDCl_3_ −75 MHz): δ (ppm) 173.71, 156.65 (C=O); 139.26, 70.19 (Cq); 126.88-128.80 (CH_Ar_); 66.83, 55.26, 53.32, 35.66 (CH_2_). UV–Visible Wavelength (nm) λ_max_: 286 in di­chloro­methane.

## 10. Refinement

Crystal data, data collection and structure refinement details are summarized in Table 4 ▸. H atoms attached to C atoms were placed in calculated positions (C—H = 0.95–0.99 Å) and were included as riding contributions with isotropic displacement parameters 1.2–1.5 times those of the attached atoms. The H atom attached to N2 was found in a difference-Fourier map and refined with a distance of 0.91 (1) Å. Two reflections, 111 and 011, affected by the beamstop were omitted from the final refinement.

## Supplementary Material

Crystal structure: contains datablock(s) global, I. DOI: 10.1107/S2056989024002445/wm5711sup1.cif

Structure factors: contains datablock(s) I. DOI: 10.1107/S2056989024002445/wm5711Isup2.hkl

Supporting information file. DOI: 10.1107/S2056989024002445/wm5711Isup3.cdx

Supporting information file. DOI: 10.1107/S2056989024002445/wm5711Isup4.cml

CCDC reference: 2340426

Additional supporting information:  crystallographic information; 3D view; checkCIF report

## Figures and Tables

**Table 1:** Hydrogen-bond geometry (Å, °) *Cg*3 is the centroid of the C4–C9 benzene ring.

*D*—H⋯*A*	*D*—H	H⋯*A*	*D*⋯*A*	*D*—H⋯*A*
N2—H2⋯O3^i^	0.91 (1)	1.95 (1)	2.8560 (15)	179 (2)
C12—H12⋯*Cg*3^ii^	0.95	2.62	3.5576 (15)	169
C18—H18*B*⋯*Cg*3^iii^	0.99	2.65	3.5726 (14)	155

Symmetry codes: (i) 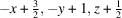 ; (ii) 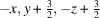 ; (iii) 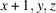 .

**Table 2:** Comparison of the selected (X-ray and DFT) geometric data (Å, °)

Bonds/angles	X-ray	B3LYP/6–311G(d,p)
O1—C2	1.2105 (15)	1.221
O2—C3	1.2183 (15)	1.223
O3—C20	1.4306 (19)	1.445
N1—C2	1.3670 (16)	1.357
N1—C3	1.4058 (16)	1.420
N2—C3	1.3485 (16)	1.338
N2—H2	0.910 (12)	0.920
N3—C17	1.4568 (16)	1.464
C20—O3—C19	110.21 (11)	111.25
C3—N2—C1	113.03 (10)	114.18
N2—C1—C4	112.19 (9)	112.69
N2—C1—C10	110.29 (9)	110.87
N2—C1—C2	100.70 (9)	100.98
O1—C2—N1	126.69 (12)	125.98
O2—C3—N2	128.46 (13)	128.52
O2—C3—N1	124.27 (12)	124.75
N2—C3—N1	107.27 (10)	107.64

**Table 3:** Calculated energies

Mol­ecular Energy (a.u.) (eV)	Compound (**I**)
Total Energy, *TE* (eV)	–32761.48
*E*_HOMO_ (eV)	–5.36
*E*_LUMO_ (eV)	–0.73
Gap, *ΔE* (eV)	4.62
Dipole moment, *μ* (Debye)	2.406
Ionization potential, *I* (eV)	–5.36
Electron affinity, *A*	–0.73
Electro negativity, *χ*	–3.04
Hardness, *η*	–2.31
Electrophilicity index, *ω*	–2.00
Softness, *σ*	0.35
Fraction of electrons transferred, *ΔN*	–2.17

**Table 4:** Experimental details

Crystal data	
Chemical formula	C_21_H_23_N_3_O_3_
*M*_r_	365.42
Crystal system, space group	Orthorhombic, *P*2_1_2_1_2_1_
Temperature (K)	150
*a*, *b*, *c* (Å)	8.4440 (3), 14.3013 (5), 15.4233 (6)
*V* (Å^3^)	1862.52 (12)
*Z*	4
Radiation type	Mo *K*α
μ (mm^−1^)	0.09
Crystal size (mm)	0.28 × 0.18 × 0.17

Data collection	
Diffractometer	Bruker D8 QUEST PHOTON 3 diffractometer
Absorption correction	Numerical (*SADABS*; Krause *et al.*, 2015 ▸)
*T*_min_, *T*_max_	0.98, 0.98
No. of measured, independent and observed [*I* > 2σ(*I*)] reflections	142315, 7105, 6502
*R*_int_	0.052
(sin θ/λ)_max_ (Å^−1^)	0.771

Refinement	
*R*[*F* ^2^ > 2σ(*F* ^2^)], *wR*(*F* ^2^), *S*	0.037, 0.097, 1.07
No. of reflections	7105
No. of parameters	247
No. of restraints	1
H-atom treatment	H atoms treated by a mixture of independent and constrained refinement
Δρ_max_, Δρ_min_ (e Å^−3^)	0.29, −0.19
Absolute structure	Flack *x* determined using 2747 quotients [(*I* ^+^)−(*I* ^−^)]/[(*I* ^+^)+(*I* ^−^)] (Parsons *et al.*, 2013 ▸)
Absolute structure parameter	0.12 (18)

Computer programs: *APEX4* and *SAINT* (Bruker, 2021 ▸), *SHELXT* (Sheldrick, 2015*a* ▸), *SHELXL* (Sheldrick, 2015*b* ▸), *DIAMOND* (Brandenburg & Putz, 2012 ▸), *SHELXTL* (Sheldrick, 2008 ▸) and *PLATON* (Spek, 2020 ▸).

## supplementary crystallographic information

### Crystal data

**Table:**

C_21_H_23_N_3_O_3_	*D*_x_ = 1.303 Mg m^−^^3^
*M**_r_* = 365.42	Mo *K*α radiation, λ = 0.71073 Å
Orthorhombic, *P*2_1_2_1_2_1_	Cell parameters from 9478 reflections
*a* = 8.4440 (3) Å	θ = 2.8–33.0°
*b* = 14.3013 (5) Å	µ = 0.09 mm^−^^1^
*c* = 15.4233 (6) Å	*T* = 150 K
*V* = 1862.52 (12) Å^3^	Block, colourless
*Z* = 4	0.28 × 0.18 × 0.17 mm
*F*(000) = 776	

### Data collection

**Table:**

Bruker D8 QUEST PHOTON 3 diffractometer	7105 independent reflections
Radiation source: fine-focus sealed tube	6502 reflections with *I* > 2σ(*I*)
Graphite monochromator	*R*_int_ = 0.052
Detector resolution: 7.3910 pixels mm^-1^	θ_max_ = 33.2°, θ_min_ = 2.8°
φ and ω scans	*h* = −13→13
Absorption correction: numerical (*SADABS*; Krause *et al.*, 2015)	*k* = −22→22
*T*_min_ = 0.98, *T*_max_ = 0.98	*l* = −23→23
142315 measured reflections	

### Refinement

**Table:**

Refinement on *F*^2^	Secondary atom site location: difference Fourier map
Least-squares matrix: full	Hydrogen site location: mixed
*R*[*F*^2^ > 2σ(*F*^2^)] = 0.037	H atoms treated by a mixture of independent and constrained refinement
*wR*(*F*^2^) = 0.097	*w* = 1/[σ^2^(*F*_o_^2^) + (0.053*P*)^2^ + 0.2332*P*] where *P* = (*F*_o_^2^ + 2*F*_c_^2^)/3
*S* = 1.07	(Δ/σ)_max_ < 0.001
7105 reflections	Δρ_max_ = 0.29 e Å^−^^3^
247 parameters	Δρ_min_ = −0.19 e Å^−^^3^
1 restraint	Absolute structure: Flack *x* determined using 2747 quotients [(*I*^+^)-(*I*^-^)]/[(*I*^+^)+(*I*^-^)] (Parsons *et al.*, 2013)
Primary atom site location: dual	Absolute structure parameter: 0.12 (18)

### Special details

**Table:**

Experimental. The diffraction data were obtained from 13 sets of frames, each of width 0.5° in ω or φ, collected with scan parameters determined by the "strategy" routine in *APEX4*. The scan time was 10 sec/frame.
Geometry. All esds (except the esd in the dihedral angle between two l.s. planes) are estimated using the full covariance matrix. The cell esds are taken into account individually in the estimation of esds in distances, angles and torsion angles; correlations between esds in cell parameters are only used when they are defined by crystal symmetry. An approximate (isotropic) treatment of cell esds is used for estimating esds involving l.s. planes.
Refinement. Refinement of F^2^ against ALL reflections. The weighted R-factor wR and goodness of fit S are based on F^2^, conventional R-factors R are based on F, with F set to zero for negative F^2^. The threshold expression of F^2^ > 2sigma(F^2^) is used only for calculating R-factors(gt) etc. and is not relevant to the choice of reflections for refinement. R-factors based on F^2^ are statistically about twice as large as those based on F, and R- factors based on ALL data will be even larger. H-atoms attached to carbon were placed in calculated positions (C—H = 0.95 - 0.99 Å) and were included as riding contributions with isotropic displacement parameters 1.2 - 1.5 times those of the attached atoms. That attached to nitrogen was placed in a location derived from a difference map and refined with a DFIX 0.91 0.01 instruction. Two reflections affected by the beamstop were omitted from the final refinement.

### Fractional atomic coordinates and isotropic or equivalent isotropic displacement parameters (Å^2^)

**Table:**

	*x*	*y*	*z*	*U*_iso_*/*U*_eq_	
O1	0.39043 (13)	0.39185 (7)	0.33591 (7)	0.0263 (2)	
O2	0.66443 (13)	0.37492 (8)	0.59106 (7)	0.0278 (2)	
O3	0.95278 (13)	0.37405 (8)	0.16772 (6)	0.0265 (2)	
N1	0.54354 (13)	0.36178 (7)	0.45643 (7)	0.01860 (19)	
N2	0.50404 (13)	0.49047 (8)	0.53375 (7)	0.01892 (19)	
H2	0.518 (2)	0.5333 (12)	0.5767 (10)	0.028*	
N3	0.80935 (12)	0.33366 (8)	0.33144 (7)	0.01897 (19)	
C1	0.42318 (14)	0.50910 (8)	0.45190 (7)	0.01579 (19)	
C2	0.44728 (14)	0.41472 (8)	0.40476 (8)	0.0177 (2)	
C3	0.58023 (14)	0.40760 (9)	0.53453 (8)	0.0191 (2)	
C4	0.24609 (14)	0.52644 (8)	0.46447 (8)	0.0175 (2)	
C5	0.16580 (16)	0.48701 (9)	0.53445 (9)	0.0226 (2)	
H5	0.222361	0.451978	0.576640	0.027*	
C6	0.00231 (18)	0.49893 (10)	0.54266 (11)	0.0296 (3)	
H6	−0.052435	0.471165	0.589914	0.036*	
C7	−0.07996 (17)	0.55110 (12)	0.48207 (12)	0.0331 (3)	
H7	−0.191083	0.559368	0.487913	0.040*	
C8	−0.00078 (18)	0.59125 (11)	0.41300 (11)	0.0308 (3)	
H8	−0.057409	0.627726	0.371901	0.037*	
C9	0.16234 (16)	0.57833 (10)	0.40347 (9)	0.0233 (2)	
H9	0.216069	0.604987	0.355331	0.028*	
C10	0.50534 (14)	0.58919 (8)	0.40337 (8)	0.01677 (19)	
C11	0.51864 (16)	0.67518 (9)	0.44551 (8)	0.0221 (2)	
H11	0.473587	0.683179	0.501458	0.026*	
C12	0.59746 (18)	0.74927 (9)	0.40608 (10)	0.0264 (3)	
H12	0.607929	0.807213	0.435648	0.032*	
C13	0.66094 (17)	0.73867 (10)	0.32348 (10)	0.0267 (3)	
H13	0.713846	0.789458	0.296334	0.032*	
C14	0.64681 (18)	0.65389 (11)	0.28097 (9)	0.0276 (3)	
H14	0.689747	0.646667	0.224429	0.033*	
C15	0.56972 (16)	0.57888 (10)	0.32074 (8)	0.0234 (2)	
H15	0.561162	0.520695	0.291395	0.028*	
C16	0.60778 (16)	0.27040 (9)	0.43367 (9)	0.0226 (2)	
H16A	0.546571	0.244049	0.384678	0.027*	
H16B	0.596317	0.227562	0.483704	0.027*	
C17	0.78214 (15)	0.27672 (9)	0.40832 (9)	0.0211 (2)	
H17A	0.842599	0.303505	0.457389	0.025*	
H17B	0.823018	0.212874	0.397594	0.025*	
C18	0.97742 (15)	0.35809 (9)	0.32419 (8)	0.0207 (2)	
H18A	1.041537	0.300511	0.318035	0.025*	
H18B	1.012307	0.391079	0.377290	0.025*	
C19	1.00178 (18)	0.42037 (10)	0.24594 (9)	0.0248 (2)	
H19A	0.940012	0.478672	0.253294	0.030*	
H19B	1.115096	0.437442	0.241412	0.030*	
C20	0.79025 (19)	0.34662 (12)	0.17402 (9)	0.0300 (3)	
H20A	0.759366	0.312437	0.120832	0.036*	
H20B	0.722946	0.403102	0.178463	0.036*	
C21	0.76244 (18)	0.28487 (11)	0.25229 (9)	0.0275 (3)	
H21A	0.648961	0.267850	0.255688	0.033*	
H21B	0.824646	0.226537	0.246373	0.033*	

### Atomic displacement parameters (Å^2^)

**Table:**

	*U*^11^	*U*^22^	*U*^33^	*U*^12^	*U*^13^	*U*^23^
O1	0.0291 (5)	0.0253 (5)	0.0245 (4)	0.0031 (4)	−0.0078 (4)	−0.0074 (4)
O2	0.0265 (5)	0.0329 (5)	0.0241 (4)	0.0036 (4)	−0.0062 (4)	0.0087 (4)
O3	0.0301 (5)	0.0309 (5)	0.0183 (4)	−0.0040 (4)	0.0023 (4)	0.0015 (4)
N1	0.0176 (4)	0.0177 (4)	0.0204 (4)	0.0023 (3)	0.0009 (4)	0.0018 (3)
N2	0.0198 (4)	0.0217 (4)	0.0153 (4)	0.0013 (4)	−0.0035 (4)	−0.0007 (3)
N3	0.0169 (4)	0.0219 (4)	0.0181 (4)	−0.0002 (4)	0.0000 (4)	0.0025 (4)
C1	0.0148 (5)	0.0178 (5)	0.0148 (4)	0.0005 (4)	−0.0015 (4)	−0.0007 (4)
C2	0.0159 (5)	0.0177 (5)	0.0193 (5)	0.0007 (4)	0.0000 (4)	−0.0009 (4)
C3	0.0158 (5)	0.0228 (5)	0.0187 (5)	−0.0012 (4)	0.0002 (4)	0.0043 (4)
C4	0.0152 (5)	0.0169 (4)	0.0202 (5)	0.0003 (4)	−0.0004 (4)	−0.0016 (4)
C5	0.0205 (5)	0.0199 (5)	0.0273 (6)	−0.0007 (4)	0.0041 (5)	0.0001 (4)
C6	0.0209 (6)	0.0281 (6)	0.0398 (8)	−0.0038 (5)	0.0087 (6)	−0.0057 (6)
C7	0.0148 (5)	0.0364 (7)	0.0480 (9)	0.0012 (5)	0.0002 (6)	−0.0126 (7)
C8	0.0211 (6)	0.0327 (7)	0.0386 (8)	0.0062 (5)	−0.0096 (6)	−0.0042 (6)
C9	0.0196 (5)	0.0252 (6)	0.0250 (5)	0.0026 (5)	−0.0048 (5)	0.0003 (5)
C10	0.0149 (4)	0.0189 (5)	0.0165 (4)	0.0008 (4)	−0.0006 (4)	0.0005 (4)
C11	0.0248 (6)	0.0200 (5)	0.0214 (5)	−0.0001 (4)	0.0027 (4)	−0.0015 (4)
C12	0.0279 (6)	0.0198 (5)	0.0315 (6)	−0.0018 (5)	0.0004 (5)	0.0020 (5)
C13	0.0214 (6)	0.0280 (6)	0.0305 (6)	−0.0008 (5)	0.0015 (5)	0.0100 (5)
C14	0.0249 (6)	0.0354 (7)	0.0225 (5)	0.0010 (5)	0.0053 (5)	0.0060 (5)
C15	0.0239 (6)	0.0274 (6)	0.0189 (5)	0.0007 (5)	0.0030 (4)	−0.0013 (4)
C16	0.0221 (6)	0.0167 (5)	0.0288 (6)	0.0022 (4)	0.0056 (5)	0.0039 (4)
C17	0.0196 (5)	0.0215 (5)	0.0223 (5)	0.0048 (4)	0.0028 (4)	0.0058 (4)
C18	0.0184 (5)	0.0246 (5)	0.0192 (5)	−0.0017 (4)	−0.0007 (4)	0.0021 (4)
C19	0.0273 (6)	0.0256 (6)	0.0214 (5)	−0.0061 (5)	0.0010 (5)	0.0020 (5)
C20	0.0290 (7)	0.0413 (8)	0.0198 (5)	−0.0061 (6)	−0.0050 (5)	0.0036 (5)
C21	0.0279 (6)	0.0321 (7)	0.0224 (6)	−0.0099 (5)	−0.0031 (5)	0.0003 (5)

### Geometric parameters (Å, º)

**Table:**

O1—C2	1.2105 (15)	C10—C15	1.3933 (17)
O2—C3	1.2183 (15)	C10—C11	1.3955 (17)
O3—C20	1.4306 (19)	C11—C12	1.3912 (19)
O3—C19	1.4373 (17)	C11—H11	0.9500
N1—C2	1.3670 (16)	C12—C13	1.390 (2)
N1—C3	1.4058 (16)	C12—H12	0.9500
N1—C16	1.4579 (16)	C13—C14	1.383 (2)
N2—C3	1.3485 (16)	C13—H13	0.9500
N2—C1	1.4597 (15)	C14—C15	1.397 (2)
N2—H2	0.910 (12)	C14—H14	0.9500
N3—C17	1.4568 (16)	C15—H15	0.9500
N3—C21	1.4608 (17)	C16—C17	1.5260 (18)
N3—C18	1.4658 (16)	C16—H16A	0.9900
C1—C4	1.5281 (16)	C16—H16B	0.9900
C1—C10	1.5342 (16)	C17—H17A	0.9900
C1—C2	1.5465 (16)	C17—H17B	0.9900
C4—C9	1.3913 (17)	C18—C19	1.5140 (18)
C4—C5	1.3938 (17)	C18—H18A	0.9900
C5—C6	1.3967 (19)	C18—H18B	0.9900
C5—H5	0.9500	C19—H19A	0.9900
C6—C7	1.383 (2)	C19—H19B	0.9900
C6—H6	0.9500	C20—C21	1.514 (2)
C7—C8	1.383 (2)	C20—H20A	0.9900
C7—H7	0.9500	C20—H20B	0.9900
C8—C9	1.397 (2)	C21—H21A	0.9900
C8—H8	0.9500	C21—H21B	0.9900
C9—H9	0.9500		
			
C20—O3—C19	110.21 (11)	C13—C12—H12	119.9
C2—N1—C3	111.86 (10)	C11—C12—H12	119.9
C2—N1—C16	125.27 (11)	C14—C13—C12	119.77 (13)
C3—N1—C16	122.83 (11)	C14—C13—H13	120.1
C3—N2—C1	113.03 (10)	C12—C13—H13	120.1
C3—N2—H2	121.6 (13)	C13—C14—C15	120.34 (13)
C1—N2—H2	124.5 (13)	C13—C14—H14	119.8
C17—N3—C21	111.74 (11)	C15—C14—H14	119.8
C17—N3—C18	110.36 (10)	C10—C15—C14	120.15 (13)
C21—N3—C18	108.22 (10)	C10—C15—H15	119.9
N2—C1—C4	112.19 (9)	C14—C15—H15	119.9
N2—C1—C10	110.29 (9)	N1—C16—C17	111.56 (11)
C4—C1—C10	112.54 (10)	N1—C16—H16A	109.3
N2—C1—C2	100.70 (9)	C17—C16—H16A	109.3
C4—C1—C2	109.27 (9)	N1—C16—H16B	109.3
C10—C1—C2	111.27 (9)	C17—C16—H16B	109.3
O1—C2—N1	126.69 (12)	H16A—C16—H16B	108.0
O1—C2—C1	126.59 (11)	N3—C17—C16	113.19 (10)
N1—C2—C1	106.71 (10)	N3—C17—H17A	108.9
O2—C3—N2	128.46 (13)	C16—C17—H17A	108.9
O2—C3—N1	124.27 (12)	N3—C17—H17B	108.9
N2—C3—N1	107.27 (10)	C16—C17—H17B	108.9
C9—C4—C5	119.48 (12)	H17A—C17—H17B	107.8
C9—C4—C1	119.88 (11)	N3—C18—C19	109.42 (11)
C5—C4—C1	120.57 (11)	N3—C18—H18A	109.8
C4—C5—C6	120.11 (13)	C19—C18—H18A	109.8
C4—C5—H5	119.9	N3—C18—H18B	109.8
C6—C5—H5	119.9	C19—C18—H18B	109.8
C7—C6—C5	120.07 (14)	H18A—C18—H18B	108.2
C7—C6—H6	120.0	O3—C19—C18	111.03 (11)
C5—C6—H6	120.0	O3—C19—H19A	109.4
C8—C7—C6	120.12 (13)	C18—C19—H19A	109.4
C8—C7—H7	119.9	O3—C19—H19B	109.4
C6—C7—H7	119.9	C18—C19—H19B	109.4
C7—C8—C9	120.18 (14)	H19A—C19—H19B	108.0
C7—C8—H8	119.9	O3—C20—C21	111.28 (12)
C9—C8—H8	119.9	O3—C20—H20A	109.4
C4—C9—C8	120.03 (14)	C21—C20—H20A	109.4
C4—C9—H9	120.0	O3—C20—H20B	109.4
C8—C9—H9	120.0	C21—C20—H20B	109.4
C15—C10—C11	119.20 (11)	H20A—C20—H20B	108.0
C15—C10—C1	122.93 (11)	N3—C21—C20	110.21 (12)
C11—C10—C1	117.85 (10)	N3—C21—H21A	109.6
C12—C11—C10	120.40 (12)	C20—C21—H21A	109.6
C12—C11—H11	119.8	N3—C21—H21B	109.6
C10—C11—H11	119.8	C20—C21—H21B	109.6
C13—C12—C11	120.12 (13)	H21A—C21—H21B	108.1
			
C3—N2—C1—C4	122.71 (11)	C5—C4—C9—C8	0.5 (2)
C3—N2—C1—C10	−110.98 (12)	C1—C4—C9—C8	177.51 (12)
C3—N2—C1—C2	6.63 (13)	C7—C8—C9—C4	−1.2 (2)
C3—N1—C2—O1	−176.25 (13)	N2—C1—C10—C15	120.60 (12)
C16—N1—C2—O1	6.2 (2)	C4—C1—C10—C15	−113.30 (13)
C3—N1—C2—C1	3.02 (13)	C2—C1—C10—C15	9.72 (16)
C16—N1—C2—C1	−174.56 (11)	N2—C1—C10—C11	−57.84 (14)
N2—C1—C2—O1	173.71 (13)	C4—C1—C10—C11	68.27 (14)
C4—C1—C2—O1	55.47 (16)	C2—C1—C10—C11	−168.71 (11)
C10—C1—C2—O1	−69.40 (16)	C15—C10—C11—C12	−1.04 (19)
N2—C1—C2—N1	−5.56 (12)	C1—C10—C11—C12	177.46 (12)
C4—C1—C2—N1	−123.80 (10)	C10—C11—C12—C13	1.3 (2)
C10—C1—C2—N1	111.33 (11)	C11—C12—C13—C14	−0.6 (2)
C1—N2—C3—O2	175.70 (13)	C12—C13—C14—C15	−0.3 (2)
C1—N2—C3—N1	−5.21 (14)	C11—C10—C15—C14	0.14 (19)
C2—N1—C3—O2	−179.73 (12)	C1—C10—C15—C14	−178.28 (12)
C16—N1—C3—O2	−2.09 (19)	C13—C14—C15—C10	0.5 (2)
C2—N1—C3—N2	1.13 (14)	C2—N1—C16—C17	103.66 (14)
C16—N1—C3—N2	178.77 (11)	C3—N1—C16—C17	−73.66 (15)
N2—C1—C4—C9	155.69 (11)	C21—N3—C17—C16	−74.95 (14)
C10—C1—C4—C9	30.62 (15)	C18—N3—C17—C16	164.58 (11)
C2—C1—C4—C9	−93.51 (13)	N1—C16—C17—N3	−62.76 (15)
N2—C1—C4—C5	−27.35 (15)	C17—N3—C18—C19	−177.76 (11)
C10—C1—C4—C5	−152.43 (11)	C21—N3—C18—C19	59.69 (14)
C2—C1—C4—C5	83.44 (13)	C20—O3—C19—C18	57.57 (16)
C9—C4—C5—C6	0.58 (19)	N3—C18—C19—O3	−59.72 (15)
C1—C4—C5—C6	−176.39 (12)	C19—O3—C20—C21	−56.62 (17)
C4—C5—C6—C7	−1.0 (2)	C17—N3—C21—C20	179.21 (12)
C5—C6—C7—C8	0.3 (2)	C18—N3—C21—C20	−59.08 (15)
C6—C7—C8—C9	0.8 (2)	O3—C20—C21—N3	58.29 (17)

### Hydrogen-bond geometry (Å, º)

*Cg*3 is the centroid of the C4–C9 benzene ring.

**Table:**

*D*—H···*A*	*D*—H	H···*A*	*D*···*A*	*D*—H···*A*
N2—H2···O3^i^	0.91 (1)	1.95 (1)	2.8560 (15)	179 (2)
C12—H12···*Cg*3^ii^	0.95	2.62	3.5576 (15)	169
C18—H18*B*···*Cg*3^iii^	0.99	2.65	3.5726 (14)	155

Symmetry codes: (i) −*x*+3/2, −*y*+1, *z*+1/2; (ii) −*x*, *y*+3/2, −*z*+3/2; (iii) *x*+1, *y*, *z*.
